# Recent Progress of Solid Lipid Nanoparticles and Nanostructured Lipid Carriers as Ocular Drug Delivery Platforms

**DOI:** 10.3390/ph16030474

**Published:** 2023-03-22

**Authors:** Viliana Gugleva, Velichka Andonova

**Affiliations:** Department of Pharmaceutical Technologies, Faculty of Pharmacy, Medical University of Varna, 55 Marin Drinov Str., 9000 Varna, Bulgaria

**Keywords:** lipid nanoparticles, mucoadhesion, ocular bioavailability, surface modification

## Abstract

Sufficient ocular bioavailability is often considered a challenge by the researchers, due to the complex structure of the eye and its protective physiological mechanisms. In addition, the low viscosity of the eye drops and the resulting short ocular residence time further contribute to the observed low drug concentration at the target site. Therefore, various drug delivery platforms are being developed to enhance ocular bioavailability, provide controlled and sustained drug release, reduce the number of applications, and maximize therapy outcomes. Solid lipid nanoparticles (SLNs) and nanostructured lipid carriers (NLCs) exhibit all these benefits, in addition to being biocompatible, biodegradable, and susceptible to sterilization and scale-up. Furthermore, their successive surface modification contributes to prolonged ocular residence time (by adding cationic compounds), enhanced penetration, and improved performance. The review highlights the salient characteristics of SLNs and NLCs concerning ocular drug delivery, and updates the research progress in this area.

## 1. Introduction

According to World Health Organization, the prevalence of eye conditions is expected to increase in the following years as a result of population aging, the associated rise of non-communicable diseases (diabetes, cardiovascular diseases), along with various lifestyle factors, such as an unhealthy diet, smoking, extensive usage of digital devices, etc. [1,2,3,4]. Furthermore, a recent analysis for the Global Burden of Disease Study forecasts that by 2050, around 474 million people will suffer from moderate to severe visual impairments, among which 61 million will develop complete blindness [5]. Although the human eye is one of the most accessible organs in terms of drug application, efficient ocular delivery is still a goal to be achieved. Possible explanations lie in the anatomical and physiological characteristics of the eyeball and its protective mechanisms, as well as in the technological properties of the ocular formulations [6]. According to location, the human eye may be distinguished into two segments: anterior, presented by the cornea, conjunctiva, iris, ciliary body, lens, and aqueous humor, and posterior, consisting of the sclera, choroid, retina, vitreous humor, and optic nerve [7,8]. The preferred route of administration in ophthalmology—topical instillation—provides the possibility for treatment of anterior segment diseases such as blepharitis, dry eye disease, conjunctivitis, ocular infections or injuries [9], however, reaching the posterior part of the eye and ensuring sufficient therapeutic concentration thereby is still a challenge. Eye drops, representing the majority of ophthalmic formulations, are relatively easy for self-administration, characterized by high patient approval, cost-effectiveness, and well-established formulation and manufacturing processes [10]. Their main limitations include their intrinsic low viscosity, a short ocular contact time, and the relatively large volume of applied drops, often leading to drug loss via physiological pathways [11,12,13].

Additionally, ocular defense mechanisms such as reflex blinking, tear turnover, nasolacrimal drainage, and static and dynamic anatomical barriers further hinder drug absorption, resulting in less than 5% of the instilled dose attaining deeper ocular tissues [14,15]. In ocular surface diseases, drug bioavailability may be partially improved through modulating the formulations’ viscosity, by including viscosity enhancers or using in situ gel-forming systems/semisolid dosage forms [16]. However, this strategy does not apply to posterior segment diseases. Unfortunately, diseases affecting the back part of the eye, e.g., age-related macular degeneration, diabetic retinopathy, and glaucoma, may often cause visual impairment or blindness unless treated efficiently [17,18]. The therapy of posterior segment eye diseases usually includes intravitreal injections, which enable drug delivery to the vitreous cavity. However, the invasive nature of this approach and the potential associated complications (e.g., endophthalmitis, retinal detachment) determine the low patient compliance [19,20]. Reaching the posterior segment via the peroral or intravenous route has also been associated with limited therapeutic success, due to the presence of blood–ocular barriers (the blood–retinal barrier, in particular), in addition to the potential risk of occurrence of side effects [21]. Altogether, these factors determine the necessity of further progress in the field of ocular delivery by improving the technological characteristics of conventional ophthalmic formulations, exploring advanced drug delivery systems, or combining both strategies.

Various nanoscale drug delivery systems, such as liposomes [22,23], niosomes [24,25], solid lipid/polymeric nanoparticles [26,27,28,29], nanostructured lipid carriers [30,31], nanomicelles [32,33], microemulsions [34,35], and dendrimers [36], have been successfully developed for ocular delivery purposes, and have been reported to achieve enhanced bioavailability, sustained and controlled drug release, and a reduction in the number of applications, as well as side effects. SLNs and NLCs raise great interest due to their excellent biocompatibility and tolerability, tunable physiochemical characteristics, and scaling-up capabilities [37,38,39]. Developed for the first in the 1990s by Professor Müller and Professor Gasco, SLNs represent a mixture of solids at ambient temperature and and lipids at physiological temperatures, dispersed in an aqueous phase containing surfactants [40,41]. Approximately 10 years later, a second generation of lipid nanoparticles was proposed—NLCs,—which additionally include liquid lipid(s) in their structure [42,43]. Both drug delivery systems are feasible carriers for hydrophilic and hydrophobic drugs. They are characterized by their long-term stability and favored uptake through biological membranes, owing to their lipid nature and nano dimensions [44,45]. The possibilities to impart mucoadhesiveness by surface coating with various polymers, or by incorporating them into semisolid/in situ gelling/formulations, further promotes their beneficial effects in ocular therapeutics.

The current review aimed to summarize the recent research progress of solid lipid nanoparticles and nanostructured lipid carriers in ocular delivery. In the first part, the anatomical and physiological features of the human eye and potential delivery routes have been discussed. The second part provides an overview of the specific characteristics of SLNs and NLCs, with respect to their compositions, suitable physicochemical properties tailored for effective ocular delivery, surface modification strategies, and sterilization feasibility. Recent advances in this area have also been outlined.

## 2. Eye Anatomy, Barriers and Routes in Ocular Drug Delivery

**Figure 1:**
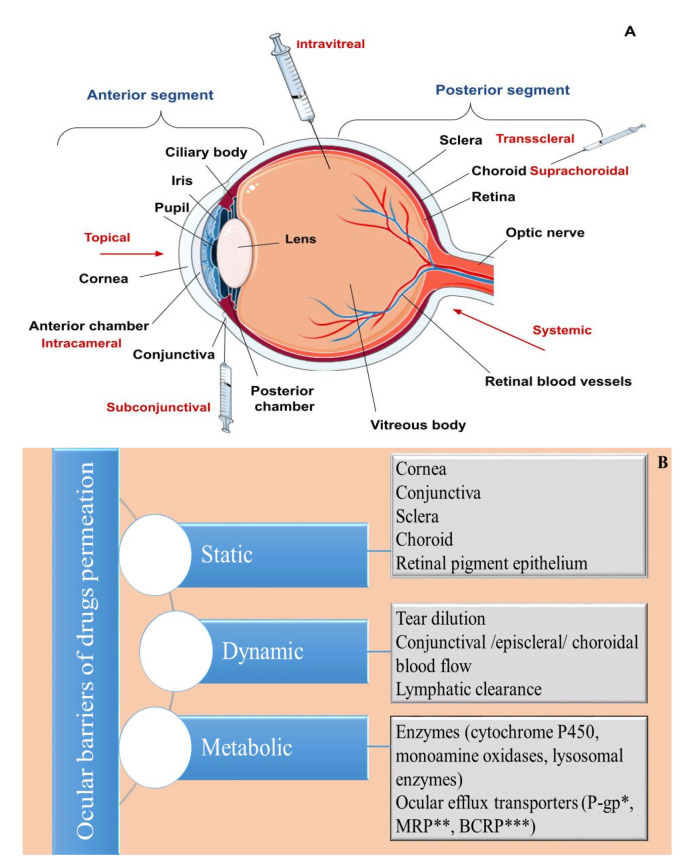
An overview of (**A**) ocular anatomy and routes for administration. (**B**) Ocular drug delivery barriers. * P-glycoprotein; ** Multidrug-resistant protein; *** Breast cancer resistance protein.

Generally, human eye structures are distinguished according to their location in the eyeball, where the eye is divided into two segments (anterior and posterior) (Figure 1A), or according to their functionalities, where it is divided into three different layers—an outer (fibrous), middle (vascular) and inner (neuronal) coat [46]. The outer layer (fibrous tunic) consists of the cornea (at its front) and sclera, occupying five-sixths of the coat [47]. Its main functions are related to maintaining the shape of the eyeball, and providing protection to the inner ocular tissues [48]. The middle layer, also referred to as uvea, is composed of the iris and the ciliary body (in the anterior), and the choroid, forming the posterior uvea (Figure 1) [49]. The retina represents the innermost layer, which is involved in the visual perception process by converting light energy into neuronal signals, which are transmitted to the visual cortex of the brain by the optic nerve [50,51].

For better perception, the anatomical and physiological features of the human eye will be discussed from the anterior to posterior segment.

### 2.1. Anterior Segment of the Eye

#### 2.1.1. Tear Film

The tear film is the first hindrance for topically applied drugs, often referred to as a dynamic (physiological) ocular barrier (Figure 1B) due to its high turnover rate, (0.5–2.2 µL/min), determining a short ocular residence time, and limited drug penetration ability [9,52,53]. Spread onto the corneal and conjunctival epithelium, it provides a smooth and lubricated optical surface, prevents the occurrence of infections due to its antimicrobial compounds (lysozyme, lactoferrin, lipocalin), or by washing out foreign substances, and supplies oxygen and nutrients to the cornea [54]. Traditionally, the tear film is described as a three-layered structure—an outer lipid layer produced by the Meibomian glands, a middle aqueous layer, and an inner mucous layer secreted predominantly by the conjunctival goblet cells [55]. However, a more recent theory considers that the tear film consists of two layers—an outer lipid layer and an inner muco-aqueous, gel-like layer [55,56,57]. Regarding ocular delivery, both layers exhibit barrier functions, the lipid one for hydrophilic drugs and the muco-aqueous layer for hydrophobic drugs [58]. Other precorneal factors negatively influencing ocular bioavailability include drug binding with proteins/mucin in the tear film, as well as drug loss via nasolacrimal drainage [53]. The latter is affected by the volume of applied drops (larger volumes correspond to more significant loss) and the blink reflex [9,12].

#### 2.1.2. Cornea

The cornea is the main route for drug absorption after topical instillation, often referred to as a static (anatomical) barrier (Figure 1B). It is a transparent, highly specialized, avascular structure comprising six layers: the corneal epithelium, Bowman’s layer, stroma, Dua’s layer, Descemet’s membrane, and endothelium [59,60]. Among these, the epithelium, the stroma, and the endothelium have a primary role in the drug/nanocarrier transport. Corneal epithelium is a five to seven-layered structure, composed of squamous, wing and basal cells [61]. Its lipophilic nature, and the existing intercellular tight junctions (zonula occludens) hinder the entry of hydrophilic substances and macromolecules [14,62]. Additionally, the presence of efflux transporters, such as breast cancer resistance protein (BCRP), multidrug resistance-associated proteins (MRPs), P-glycoprotein (P-gp), and enzymes (e.g., cytochrome P450), acting as metabolic barriers, may further decrease ocular drug bioavailability [58,63,64]. Beneath the epithelium is the stroma, which occupies approximately 90% of the corneal thickness [65]. It is a hydrophilic, gel-like structure made of collagen fibrils and mucopolysaccharides, and represents the main obstacle for the permeation of lipophilic compounds [66]. The corneal endothelium is a single layer composed of hexagonal-shaped cells involved in water transport towards the anterior chamber, as well as the maintenance of corneal transparency [67]. Unlike the epithelial layer, the endothelial junctions are considered “leaky” and enable the transport of macromolecules [11]. In general, drugs are transported across the cornea via transcellular (for lipophilic compounds) and paracellular (for hydrophilic molecules) pathways [68]. Factors affecting corneal absorption include a drug’s molecular weight (compounds up to 500 Da are able to permeate across the epithelium), lipophilicity (facilitated for lipophilic compounds; preferably log D values of 2–3), degree of ionization (non-ionized forms penetrate more easily), and the charge of the ionized species (facilitated penetration of cationic molecules) [21,69,70,71].

#### 2.1.3. Conjunctiva

The conjunctiva is a transparent mucous membrane, which overlays the anterior ocular surface and the interior of the eyelids. It is involved in the production of mucus and the maintenance of the tear film, ensuring the lubrication of the eye, and also preventing the entrance of exogenous substances or microorganisms [53,72]. The conjunctiva may be divided into three areas: the bulbar conjunctiva, covering the anterior part of the sclera; the conjunctival fornices, forming the cul-de-sac; the palpebral conjunctiva located on the posterior eyelid’s surface [50]. Generally, the cul-de-sac is estimated to retain a volume of up to 30 µL—a capacity insufficient to preserve the entire volume of an applied drop (most often in the range of 40–70 µL), which leads to partial drug loss immediately after instillation [67]. The conjunctiva is considered to be more permeable when compared to the cornea, especially in terms of hydrophilic compounds, due to the wider intercellular spaces between the junctions in its structure, allowing for the passage of larger compounds (5000–10,000 Da), as well as owing to its bigger surface area. Nevertheless, conjunctival drug absorption is considered ineffective, mainly due to its high vascularity [71,73,74]. Conjunctival blood and lymph circulation functions as a dynamic barrier, leading to drug clearance and systemic absorption, hence the observed low drug concentration in the anterior chamber. Additionally, the existing transporters (amino acids transporters, P-gp) acting as efflux pumps further contribute to this process [63,75].

#### 2.1.4. Iris

The iris is a circular, colored, contractile structure, which surrounds an aperture in its center (the pupil) (Figure 1A). It regulates the constriction or dilation of the pupil according to the light intensity, via parasympathetic/sympathetic activation, respectively [76]. It contains pigmented epithelial cells in its structure, enabling drug accumulation and altering its pharmacokinetics [77]. The melanin-containing cells in the eye (localized to the iris/ciliary body at the front and in the choroid/retinal pigment epithelium in the posterior) can bind drug molecules via electrostatic and van der Waals forces, as well as by charge interactions. The formed complex may be considered a “reservoir”, releasing drugs at a slow rate, therefore, it can also be used in a drug-targeting approach to achieve prolonged action in the corresponding (pigmented) ocular areas [78,79,80].

#### 2.1.5. Ciliary Body

The ciliary body is part of the middle (vascular) layer in the eye and is involved in the maintenance of the shape of the lens via the ciliary muscle, and in the production of aqueous humor [53,81]. Furthermore, the ciliary epithelium and the endothelial cells of the iris blood vessels form the *blood–aqueous barrier (BAB)*, which prevents molecules’ entrance from systemic circulation to the aqueous humor [82]. The tight junctions in its structure limit the paracellular transport of large hydrophilic molecules, unlike small lipophilic compounds, which can penetrate via the transcellular pathway, and are subsequently eliminated by the uveal blood flow and aqueous humor turnover [49,78,83,84]. Alternatively, the elimination of hydrophilic compounds from the anterior chamber is carried out solely by the aqueous humor through Schlemm’s canal, which determines their slower clearance [67,78].

#### 2.1.6. Lens

The lens is located behind the iris and the pupil (Figure 1A), and is characterized by its transparent appearance, biconvex shape, great index of refraction, and high concentration of proteins in its structure (i.e., crystallins). Its main functions include light transmission and focusing it onto the retina to obtain a distinct image [85,86].

### 2.2. Posterior Segment of the Eye

The sclera, the choroid, and the retinal pigment epithelium (RPE) represent the posterior static ocular barriers used for drug delivery [63].

#### 2.2.1. Sclera

The sclera is a white, dense tissue, made of collagen fibers (predominantly type I, and <5% type III) and proteoglycans [87]. The porous areas within the collagenous, aqueous medium determine the relatively easy passage of hydrophilic molecules when compared to hydrophobic ones. In addition to drugs’ lipo/hydrophilicity, other physicochemical characteristics, such as their charge, molecular weight, and molecular radius, also influence scleral permeability [19]. The proteoglycan matrix, negatively charged at physiological pH, hinders the permeation of positively charged compounds as a result of the electrostatic interactions in between [88]. Regarding the impact of molecular weight/radius, studies showed that molecules up to 70 kDa are able to permeate across the sclera [89], and there is an inverse relationship between radius and drug permeability—smaller molecules penetrate more easily [88].

#### 2.2.2. Choroid

The choroid is a thin, vascularized, pigmented tissue, involved in the transport of nutrients and oxygen to the retina [90,91]. Concerning drug delivery, it may be considered as both a static and dynamic barrier (Figure 1B), the latter owing to its high blood flow rate, determining rapid drug elimination [7,92]. Choroidal blood vessels are characterized by fenestrated walls, which enable drugs to reach the extravascular space of the choroid. Still, their further distribution towards the retina is limited by the presence of the blood–retinal barrier (BRB) [14,78].

#### 2.2.3. Retina

The retina is a thin, transparent tissue lining the inner ocular surface [50]. It is characterized by a complex structure—histologically, it can be divided into ten layers. The outermost layer, the retinal pigment epithelium, represents a significant barrier to ocular drug delivery, due to the existing tight junctions between the epithelial cells, hindering paracellular drug transport [93,94]. The retinal pigment epithelium participates in the formation of the *blood–retinal barrier* (the outer BRB), whereas the retinal capillary endothelial cells constitute the inner BRB [95].

#### 2.2.4. Vitreous Body

The vitreous body is a clear, avascular gel-like substance occupying the majority of the eyeball (Figure 1A) [96]. It performs several important functions, including maintaining the shape of the eyeball, acting as a shock absorber, protecting the retina from mechanical stress, and participating in light transmission towards the retina [97]. The vitreous body may be also considered as an area for drug delivery to the posterior eye segment. Intravitreal permeation depends on drugs’ physicochemical characteristics, such as their charge (facilitated for negatively charged molecules, which do not interact electrostatically with the negatively charged vitreous humor constituents), size (small molecules diffuse easily), and lipophilicity (easier when compared to hydrophilic drugs). The last two parameters also influence drug clearance—larger and hydrophilic molecules are characterized by a longer half-life, due to their elimination via the anterior route (through the aqueous humor), in contrast to small lipophilic compounds, which are cleared via the posterior route (crossing the BRB) [19,21,69].

### 2.3. Alternative Routes of Ocular Delivery

The complex anatomical and physiological features of the eye elucidate the challenges in ocular drug delivery from a physiological point of view. To achieve higher therapeutic concentrations in the posterior segment, alternative routes of administration have been exploited, the most common of which are presented in Table 1. However, most of them (excluding the oral route) are invasive, and are not applicable by the patients themselves, therefore, research efforts are focused on the elaboration of advanced drug delivery platforms, aiming to improve drug bioavailability and therapeutic outcomes for both anterior and posterior eye segment diseases.

## 3. Feasibility of Lipid Nanoparticles in Ophthalmology

Lipid-based drug delivery systems, such as nanoemulsions, liposomes, niosomes, cubosomes, and lipid nanoparticles, have attracted an enormous scientific interest, due to their biocompatibility, biodegradability, and tolerability [108]. An excellent review summarizing the feasibility of all the aforementioned lipid-based nanocarriers in ophthalmology is provided here [109]. Emerging initially as an alternative to liposomes in terms of their superior physical stability, cost-effective process and materials, as well as being alternatives to polymeric nanoparticles, due to the absence of toxic degradation products, [37] SLNs have been explored as drug delivery systems for various routes of application—dermal [110,111], ocular [112,113], pulmonary [114], parenteral [115], nasal [116], and oral [117]. Another advantageous characteristic of the lipid nanocarriers is the possibility of encapsulating more than one therapeutic agent, leading to the elaboration of dual or multidrug lipid nanoparticles, characterized by a synergetic effect and improved therapeutic performance [118]. In ophthalmology, in particular, SLNs and the second-generation lipid particles—NLCs—are considered especially beneficial due to their ability to provide sustained drug release by acting as drug depot formulations, and enhance corneal penetration due to the corresponding activity of non-ionic surfactants included in their structure [119,120]. The latter may further contribute towards an improved ocular bioavailability, by opening the tight junctions between corneal epithelial cells, facilitating paracellular drug transport, and by inhibiting P-glycoprotein activity, limiting drug efflux [121,122,123].

The lipid nanoparticles’ transcorneal penetration mechanism has been studied by Nagai et al., according to which the process is implemented via energy-dependent endocytosis. The authors proposed three endocytosis pathways (clathrin-dependent, caveolae-dependent and macropinocytosis) as possible mechanisms for penetration of indomethacin-loaded nanoparticles, with an emphasis on the caveolae-dependent endocytosis [124]. Undoubtedly, nanoparticles’ permeation and internalization are highly affected by their physicochemical characteristics, such as size, size distribution pattern, zeta potential, and subsequent surface modification. Generally, nanoparticles up to 200 nm are reported to penetrate across the cornea [125]. In the case of periocular application, the excessive downsizing of their dimensions (e.g., ≈20 nm) may lead to their rapid clearance, as reported by Amritte et al. [126]. In their study Niamprem et al. investigated the penetration of fluorescent dye (Nile red)-loaded NLCs across porcine cornea, as a function of their size and surface modifications. According to the authors, NLCs with a size of 40 nm exhibited enhanced penetration when compared to larger (150 nm) nanoparticles.

Regarding their internalization, non-modified NLCs had a higher uptake in porcine corneal epithelial cells than PEG- and stearylamine-modified nanocarriers. The latter may be attributed to their superior mucoadhesive properties, arising from hydrogen boding between PEG molecules and mucin glycoproteins, or from ionic interactions between cationic stearylamine and anionic groups present in mucin regions [127].

Ocular drug delivery is also affected by the zeta potential of the nanocarriers. Positive values contribute to an increased ocular contact time, as a result of the occurred electrostatic interactions with the negatively charged corneal epithelium [125]. Regarding zeta potential’s impact on the colloidal stability of the nanocarriers, generally, absolute values of 30 mV are considered to be sufficient to provide repulsion between the nanoparticles in the dispersion and prevent their aggregation [128].

### 3.1. Lipid Nanoparticles—Structural Features and Recent Progress in Ocular Therapeutics

**Figure 2:**
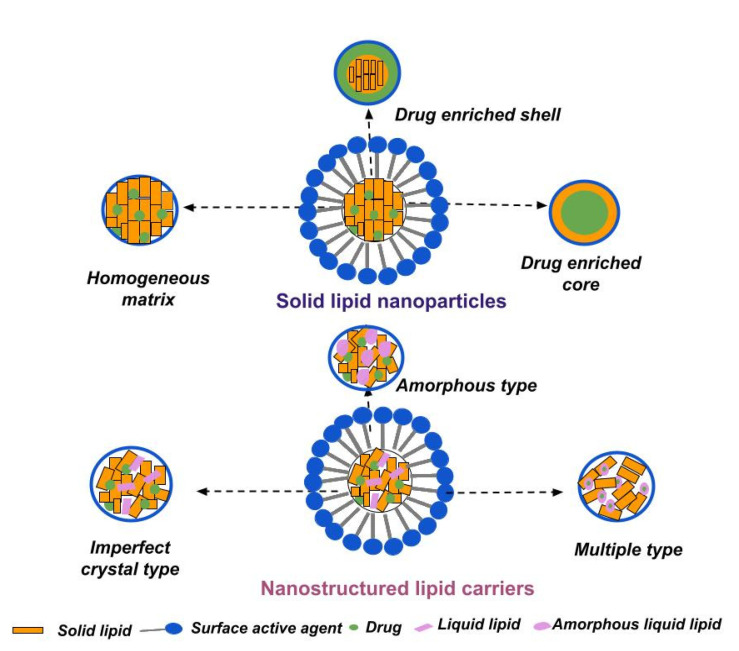
Different types of solid lipid nanoparticles and nanostructured lipid carriers.

According to their main structural components, lipid nanoparticles may be distinguished into solid lipid nanoparticles (composed of solid-state lipids under ambient and physiological conditions) and nanostructured lipid carriers (additionally containing liquid lipids in their composition). In both cases, the lipid constituents are dispersed in an aqueous medium stabilized by surfactants [108]. Their specific structures and types are illustrated in Figure 2.

#### 3.1.1. Solid Lipid Nanoparticles

Solid lipid nanoparticles are generally sphere-shaped colloidal systems, ranging between 50 and 1000 nm, and have been successfully explored as carriers for both hydrophilic and hydrophobic drugs [129]. The most frequently used solid lipids for their preparation include *triglycerides* (tristearin (Dynasan 118), tripalmitin (Dynasan 116), trimyristin (Dynasan 114)), a *mixture or mixtures of mono-, di- and triglycerides* (glyceryl behenate (Compritol 888 ATO), glyceryl palmitostearate (Precirol ATO 5)), *waxes* (beeswax, carnauba wax), *fatty acids* (lauric/stearic/myristic acid), and the corresponding *fatty alcohols* [130,131].

The chemical structure of lipids has a major impact on their physicochemical properties and delivery process of the nanoparticles, as reported by several studies. Boonme et al. investigated the effect of different lipids (glyceryl trimyristate, glyceryl tripalmitate, glyceryl tristearate, stearic acid, glyceryl monostearate) on the characteristics of SLNs obtained by the microemulsion technique. The selected lipids differ in the number of C atoms of the fatty acids chains, as well as their polarity. According to the obtained results, lipid polarity influences the capability to obtain microemulsions—the formation of such was reported in three of the studied formulations (comprising glyceryl monostearate, stearic acid and glyceryl trimyristate). This may be related to the absence of polar functional groups in the structure of glyceryl tripalmitate/glyceryl tristearate, as well as to their long (C-16/C-18) chains, determining large molecular volumes unable to penetrate into the hydrophobic region of the surfactant interface. The number of carbon atoms of the fatty acid residue also affects nanoparticle size—the smallest diameter was observed in the glyceryl trimyristate-based formulation, as a result of the shorter carbon chain (14 C atoms vs. C18 atoms) facilitating its penetration into the surfactant’s interface [132]. Palival et al. investigated the influence of several solid lipids (stearic acid, glycerol monostearate, tristearin, and Compritol 888 ATO) on the properties of methotrexate-loaded SLNs intended for oral delivery. According to the obtained results, the highest entrapment efficacy was reported for the Compritol 888-based SLNs, which may be related to the drug interchain intercalation [133].

The appropriate selection of a solid lipid or lipid mixture is an important subject, as it impacts the physicochemical characteristics (size, drug loading capacity), as well as drug release and storage stability, of the nanocarriers. Important issues to be considered during (pre)formulation studies include the solubility of drug in the lipid matrix, drug/lipid compatibility, and the lipid(s) crystalline behavior [134,135]. Based on the structural organization and drug location within the nanoparticles, three types of SLNs can be distinguished, as illustrated in Figure 2.

The *homogenous matrix model* is characterized by a uniformly allocated drug within the lipid matrix (molecularly dissolved or in form of amorphous clusters), mainly produced via the high-pressure homogenization method. The homogenous matrix particles result from the agitation of the dispersed drug in bulk lipid (when the cold technique is applied) or from the crystallization of cooled liquid droplets, in the case of hot homogenization. The latter is suitable for highly lipophilic drugs, without the necessity of using solubilizing agents [136].

The *drug-enriched shell model* involves predominantly localizing the drug in the outer shell of the nanoparticles, arising from phase separation and drug migration during the cooling stage of the process. Fast cooling induces the lipid in the center to precipitate, whereas the drug concentration in the residual liquid lipid increases, forming the outer shell. This model is characterized by fast drug release [137].

The *drug-enriched core model* is characterized by a high drug concentration in the melted lipid, leading to supersaturation of the drug and its precipitation during the cooling phase before lipid recrystallization. Further cooling subsequently leads to lipid recrystallization, and to the formation of a membrane overlaying the drug-enriched core [138].

In addition to the lipid constituents, a SLN formulation also contains surfactants, which facilitate the dispersion of lipids within the aqueous medium and stabilize the system by reducing the interfacial tension between both immiscible phases [139]. Generally, surfactants are included in the composition up to 5%*w/w*, and their selection is based upon several considerations, such as hydrophilic–lipophilic balance (HLB value), the route of administration of SLNs, safety profile, and compatibility with the other excipients [135,140]. In SLNs, intended for ophthalmic applications, the most-often included surfactants are non-ionic, such as polyoxyethylene sorbitan fatty acid esters (Polysorbates/Tweens), polyoxyethylene/polyoxypropylene block copolymers (Poloxamers/Pluronic), and amphoteric molecules, e.g., soy lecithin, due to their superior safety profiles compared to their anionic or cationic counterparts [119,131].

In their study, Silva et al., 2019 investigated the cytotoxicity of SLNs, containing the cationic surfactants cetyltrimethylammonium bromide (CTAB) and dimethyldioctadecylammonium bromide (DDAB), against five human cell lines of different origin. According to the obtained results CTAB-containing SLNs exhibited superior cytotoxicity in comparison to DDAB-SLNs, as the experimental concentration is closer to the critical micellar concentration of CTAB (the latter is related to cell lysis) [141].

SLNs may also contain cryoprotectants (e.g., trehalose, sorbitol, mannitol), in case the nanoparticles are subjected to lyophilization [142], as well as surface-modifying additives, such as polyethylene glycol, to confer stealth properties of the nanocarriers [143], or selective ligands, antibodies, etc., to provide targeted delivery [144,145]. In ocular therapeutics, SLNs are often modified using polyethylene glycol to improve their pharmacokinetic profile, or are coated with mucoadhesive polymers (e.g., chitosan), aiming to prolong their precorneal residence time [146,147].

In their study, Eid et al. investigated the impact of PEGylation and chitosan coating on the ocular bioavailability of ofloxacin-loaded SLNs. The addition of PEG stearate to the compositions determined higher transcorneal permeability, with a moderate effect on the mucoadhesion, in contrast to chitosan, which exerted the opposite effects. Ultimately, the developed PEGylated chitosan-coated SLNs improved the ocular bioavailability of ofloxacin by increasing the drug concentration in rabbits’ eyes two- to three-fold when compared to the plain drug [148]. The PEGylation approach was also adopted by Dang et al., who developed a PEGylated SLNs-laden contact lens, characterized by an enhanced latanoprost-loading capacity, smaller sizes (compared to non-PEGylated SLNs), and sustained drug release up to 96 h [149].

The development of *hybrid drug-delivery platforms* based on nanocarriers and a vehicle (semisolid formulations, in situ gels, contact lens) is an advantageous strategy for ocular delivery purposes, as it exploits the beneficial effects of both systems. In their study, Sun and Hu developed tacrolimus-loaded SLNs that were thermosensitive in situ gel, which were characterized by suitable gelling and rheological characteristics (gelation temperature 32 °C, pseudoplastic behavior), sustained drug release and improved pharmacodynamic effects when compared to the free drug and tacrolimus-loaded SLNs [150]. Improved biopharmaceutical and therapeutic outcomes were reported also for mizolastine-loaded hydrogel SLNs, manifesting in sustained drug release (up to 30 h) and reduced symptoms of allergic conjunctivitis in rabbits’ eyes [151].

Another beneficial SLN-based delivery strategy implemented in ocular therapeutics is the elaboration of dual solid lipid nanoparticles, as reported by Carbone et al. [152]. The authors aimed to improve the effectiveness of *Candida albicans* mycosis treatment by combining the antimycotic effect of clotrimazole and the antioxidant activity of alpha-lipoic acid. SLN as a delivery platform enabled the simultaneous loading of both drugs, and determined slow and controlled drug release, without an initial burst effect. The latter was achieved due to the successful incorporation of both drugs within the inner lipid matrix, and not on the nanoparticles’ surface [152].

An overview of the developed SLNs for ocular delivery purposes is provided in Table 2.

As presented in Table 2, SLNs have been successfully exploited for both anterior and posterior eye segment diseases. The reported therapeutic results may be attributed to various factors, such as the ability of SLNs to form a depot for the prolonged release of the drug, the fluidizing effect of included surfactants on the lipid bilayers of ocular membranes, facilitating drug permeation, as well as the large surface area of nanocarriers, providing maximized contact with the ocular mucosa [163,164]. It is also worth noting the ability of SLNs to encapsulate high molecular weight compounds, such as atorvastatin [158] and natamycin [154], which are also characterized by poor solubility, therefore, their ocular delivery through conventional ophthalmic formulations would be a challenge. Encapsulation of atorvastatin in SLNs further contributed to improved drug photostability, as confirmed by the photostability studies conducted according to ICH guidelines [158]. Liang et al. also reported overcoming the unfavorable characteristics of the drug by developing econazole-loaded SLNs. The antimycotic is characterized by low aqueous solubility and strong irritation potential, which restrain its application in the therapy of ocular fungal infections. The conducted in vivo studies showed enhanced corneal permeation, and no ocular irritation with the econazole-loaded SLNs [153]. Solid lipid nanoparticles are also beneficial in the therapy of posterior segment diseases, e.g., glaucoma, as confirmed by the superior intraocular pressure reduction [162], and higher therapeutic concentration in the iris, ciliary body, and retina [163].

The pre-clinical safety of SLNs was evaluated in polymeric nanospheres and liposomes in a recent study conducted by Gomes Souza et al. The authors elaborated sunitinib-loaded nanocarriers as topical formulation strategies for corneal neovascularization treatment. The sunitinib-loaded SLNs were selected as the optimal formulation due to their excellent tolerability profile, controlled drug release, and highest corneal retention [165].

#### 3.1.2. Nanostructured Lipid Carriers

Nanostructured lipid carriers were initially developed to surmount the limitations associated with SLNs, such as their poor drug-loading capacity, owing to their perfectly arranged crystalline structure, and their propensity towards drug expulsion during storage, resulting from lipid crystallization [37,166]. The addition of spatially incompatible liquid lipid(s) to the formulations is beneficial in two aspects, it leads to the formation of a less-ordered crystalline structure (Figure 2), ensuring extra area for drug loading, decreases the crystalline degree of the lipid matrix and averts drug expulsion [128,167]. Usually, the liquid lipid is included up to 30% of the total lipid amount in the NLCs formulations [168,169]. As such, researchers often use castor/olive/argan oil, oleic acid, Miglyol^®^ 812 (medium-chain triglycerides), propylene glycol dicaprylocaprate—Labrafac™ PG (Gattefosse, Saint-Priest, France), or caprylocaproyl macrogol-8 glycerides—Labrasol^®^ (Gattefosse, Saint-Priest, France) [170,171,172].

The selection of both solid and liquid lipids is reported to influence NLCs’ size. According to Apostolou et al., NLCs comprising solid lipids, such as Precirol ATO 5 (Gattefosse, Saint-Priest, France), Compritol 888 ATO (Gattefosse, Saint-Priest, France) or Dynasan 118 (IOI Oleo GmbH, Hamburg, Germany), exhibit larger particle sizes when compared to glyceryl monostearate- and stearic acid-based nanocarriers. A possible explanation may lie in the higher molecular weight of the lipids, leading to the formation of a more complex structure, with a tendency of aggregation between the molecules, which results in an increased nanoparticle diameter [173]. Concerning the selection of liquid lipids, NLCs containing Mygliol^®^ 812 (IOI Oleo GmbH, Hamburg, Germany) are generally characterized by larger size when compared to oleic acid or Capryol 90-containing ones (Gattefosse, Saint-Priest, France) [174,175,176].

NLCs can be classified into three models depending on the preparation methods, lipid matrix structure, and drug location [177].

The ***imperfect type*** is obtained by blending structurally different lipids, resulting in the formation of disorganized lipid matrix. The selected lipids, usually a small fraction of liquid oil mixed with larger amount of solid lipid, may differ in terms of fatty acid origin, in their carbon chain length or degree of saturation. This type of NLC is characterized by its high drug-loading capacity, proportionally related to the imperfections within the lipid matrix [178].

The ***amorphous-type*** NLCs are formed owing to the addition of specific lipids to the formulation, such as hydroxyoctacosanyl hydroxystearate and isopropyl myristate. These lipids contribute to the formation of a non-crystalline (amorphous) matrix, limiting drug expulsion as a result of solid lipid crystallization [143].

The ***multiple-type*** NLCs are oil-in-solid, fat-in-water nanocarriers, composed of numerous liquid oil nanocompartments within a solid lipid matrix, usually obtained through the hot homogenization technique. The greater amount of liquid lipid in the formulation leads to phase separation and the formation of the nanosized droplets upon the cooling phase. The multiple-type NLCs are characterized by high drug-loading capacities, due to the superior solubility of lipophilic drugs in liquid lipids compared to those in solid ones. Furthermore, the solid matrix exhibits a barrier function, limiting drug leakage and controlling the release process [178,179].

Similar to SLNs, the surface of NLCs can be modified with cationic additives (e.g., chitosan) to impart muco-adhesiveness, sustained drug release, and increased penetration, as reported by Selvaraj et al. [180], Sharma et al. [181], and Fu et al. [182]. Derivatives of chitosan (trimethyl chitosan) and chitin (chitosan oligosaccharide) have also been investigated as nanoparticle surface-coating materials, as they exhibit improved aqueous solubility at a neutral pH (including in the lacrimal fluid) and superior safety profiles compared to native chitosan, while at the same time retaining all of its beneficial characteristics (biodegradability, muco-adhesion, penetration-enhancing properties, etc.) [183,184].

Mucoadhesive NLCs have also been developed by functionalization with (3-aminomethylphenyl) boronic acid attached to chondroitin sulfate, to increase corneal residence time by specifically targeting the sialic acid residues on the ocular surface, which ultimately improves drug performance regarding dry eye disease [185]. In vivo relief of dry eye disease symptoms, accompanied by enhanced corneal retention, was also reported by Zhu et al., developing chondroitin sulfate and L-cysteine conjugate-modified dexamethasone NLCs [186].

In another study Abdelhakeem et al. elaborated on surface-modified eplerenone-loaded NLCs for the treatment of central serous chorioretinopathy. The authors evaluated the effect of three different coating polymers (hyaluronic acid, chitosan oligosaccharide lactate, and hydrogenated collagen) on the properties of the nanocarriers. The largest particle size was reported for the hyaluronic acid-coated NLCs, corresponding to the formulation’s highest eplerenone entrapment efficiency and viscosity. The higher viscosity determined the superior sustained drug release from hyaluronic acid-modified NLCs compared to the other NLCs models. The selected optimal formulations (hyaluronic acid/chitosan oligosaccharide lactate-coated) were characterized by an excellent ocular tolerability, as confirmed by the Draize test [187].

Nanostructured lipid carriers have been also an integral component of ***hybrid drug-delivery platforms***, recently included into thermosensitive in situ gel-forming systems [188,189]. An interesting approach is described by Yu et al. in two of their studies, elaborating on baicalin NLCs and quercetin NLCs that were subsequently incorporated into dual pH and thermosensitive in situ gels. The dual stimuli-responsive formulation was based on carboxymethyl chitosan and Poloxamer 407, cross-linked by the natural cross-linker genipin. Both hybrid, NLC-loaded, in situ gels were characterized by prolonged drug release and precorneal residence time, and improved transcorneal penetration compared to eye drops [190,191].

***Dual nanostructured lipid carriers*** have also been developed for ocular delivery purposes. In their study Youseff et al. developed simultaneously loaded natamycin/ciprofloxacin NLCs as a drug delivery system for microbial keratitis treatment. The selection of model drugs (an antifungal agent and fluoroquinolone antibiotic) was based on the complex etiology of corneal infections (which may be caused by bacteria/fungi/protozoa, when a secondary or co-infection is present). The elaborated dual NLCs were subsequently incorporated into in situ ionic gel formulations, aiming to further enhance the therapeutic efficacy by providing prolonged ocular surface contact time [120]. Dual therapeutic synergy was exploited also by Chen and Wu when developing brinzolamide- and latanoprost-loaded NLCs for the therapy of glaucoma (details of the study are presented in Table 3) [192].

Further overview of the recent progress of NLCs in ocular therapeutics is shown in Table 3.

Nanostructured lipid carriers are feasible delivery systems for both drugs and biologically active compounds, as illustrated in Table 3. Polyphenolic compounds are well-known for their antioxidant effects, which would be highly beneficial in the therapy of ocular degenerative diseases. However, these phytochemicals are usually characterized by poor aqueous solubility and an unfavorable pharmacokinetic profile, as reported for diosmin [203] and mangiferin [204]. Their encapsulation in NLCs led to an improvement of their disadvantageous physicochemical properties (e.g., low aqueous solubility), and further contributed to superior antioxidant activity (in the case of the mangiferin-loaded NLCs) and cytoprotective effects (for diosmin-loaded NLCs). Other beneficial outcomes following drug loading into NLCs include superior chemical/photo stability, estimated by the rapamycin-loaded NLCs [195], as well as the pronounced enhancement of the solubility of dasatinib upon encapsulation. The latter further contributes to the observed higher anti-proliferation and anti-migration effects [198].

In addition to the conventional topical application, NLCs have been formulated for periocular administration (transscleral delivery), as reported by González-Fernández et al. The authors prepared dexamethasone acetate-loaded NLCs intended for the treatment of posterior eye segment diseases (e.g., macular edema, age-related macular degeneration). The encapsulated prodrug acetate ester provided sustained drug release as a result of the required enzymatic conversion step, and enhanced scleral/choroidal permeability [205].

### 3.2. Sterilization Feasibility of SLNs and NLCs

Owing to their compositional similarities, NLCs and SLNs can be prepared by identical methods, such as high-pressure homogenization (hot/cold option), high-speed homogenization and/or ultrasonication, solvent emulsification/evaporation, microemulsion, phase inversion techniques, and the solvent injection method [143]. A comprehensive description of the various preparation methods has been detailed by Gordillo-Galeano and Mora-Huertas [131], Khairnar et al. [206] and Duong et al. [207]. However, of great importance for ocular application is one of the post-production steps, namely, the sterilization feasibility.

Techniques such as heat sterilization (autoclaving), sterile filtration and gamma irradiation have been used as ***sterilization methods*** for SLNs and NLCs intended for ophthalmic application. The selection of the specific method is based on several considerations, such as drug heat stability, composition constituents (melting point of lipids, choice of surfactants), nanoparticle size, and the viscosity of the solution in case of sterile filtration [83,162,208]. ***Autoclaving*** is the most commonly exploited technique for the sterilization of lipid nanoparticles in ophthalmology, however, with controversial results regarding its impact on the physiochemical characteristics of the nanocarriers. According to some reports, there is no significant change in the particle size [158,172,209] or entrapment efficiency [158] of developed lipid nanocarriers before and after sterilization, in contrast to others, which established an increase in particle size in the micrometer range [210]. The latter may be ascribed to the compromised surfactant film properties, as well as to the melting of lipids at 121 °C, leading to the formation of an o/w emulsion. During the successive cooling and lipid recrystallization, no energy input (i.e., homogenization) was applied to the system, resulting in the increase of particle size [210]. In their study Youshia et al. investigated the influence of autoclaving and ***sterilization by gamma irradiation*** on the physicochemical parameters of methazolamide-loaded cationic NLCs. According to the results, NLCs subjected to heat sterilization were characterized by significantly lower entrapment efficiency and zeta potential values. At the same time an increase in the particle size and polydispersity index was observed. On the contrary, gamma radiation did not induce significant alterations in the particles size, size distribution pattern, or in the degree of methazolamide entrapment [211]. However, one of the main limitations of this method is the formation of free radicals, therefore, subsequent studies need to be performed, in order to evaluate the chemical stability of the components. Additionally, different strategies may be applied to mitigate the adverse effects of radiation, such as adjustment of the applied dose, lyophilization of the samples, and the use of suitable (endure to γ-radiation) excipients [208].

***Sterile filtration*** has also been exploited as a sterilization approach for lipid nanoparticles used in ophthalmic application, as described by Bonaccorso et al. [157]. The authors investigated the influence of different types of membranes (polypropylene, polyethylene sulfone, polyvinylidene fluoride; pore size of 0.22 µm) on the filtration feasibility of sorafenib-loaded SLNs. The obtained results showed that polypropylene and polyethylene sulfone filters restrain the filtration process by retaining the nanoparticles within the membrane, unlike the polyvinylidene fluoride membrane, which enables SLNs’ passage. Furthermore, the obtained SLN suspension after filtration was characterized by unaltered physiochemical parameters [157].

### 3.3. Clinical Application of SLNs and NLCs in Ocular Therapeutics

Several lipid-based ophthalmic nanocarriers have been successfully implemented into clinical practice, such as Visudyne^®^ (Novartis Pharma AG, Basel, Switzerland), a liposomal verteporfine nanoformulation intended for the therapy of age-related macular degeneration, Durezol^®^ (Alcon, Geneva, Switzerland), a difluprednate nanoemulsion for ocular inflammation treatment, and Restasis^®^ (AbbVie, North Chicago, IL, USA), a cyclosporine nanoemulsion intended for the therapy of dry eye disease [212,213]. However, regardless of the positive outcomes garnered from conducted studies, currently, there are no SLN- or NLC-based ophthalmic formulations that have been translated into clinical applications or marketed. A search through the website www.clinicaltrials.gov (accessed on 1 March 2023) using the keyword ”solid lipid nanoparticles” resulted in 10 studies, whereas the keyword “nanostructured lipid carriers” led to 2 results. Currently, none of these trials are related to ocular delivery purposes. Further details are provided in Table S1.

## 4. Conclusions and Prospects

Solid lipid nanoparticles and nanostructured lipid carriers have shown significant potential for effective ocular drug delivery, as confirmed by the findings summarized in this review. Their advantageous characteristics such as biodegradability, biocompatibility, owing to the generally recognized as safe (GRAS) lipid constituents, and their possibility to provide controlled and sustained drug release, to improve transcorneal penetration and enhance ocular bioavailability, determine their increasing progress in ocular therapeutics. Furthermore, the surface of both types of nanocarriers can be modified to improve their pharmacokinetic characteristics, impart mucoadhesive properties, prolong corneal residence time, and enhance their therapeutic efficacy. The latter can also be achieved by incorporating them into semisolid/in situ gelling formulations and contact lenses (i.e., hybrid delivery systems), which is another promising research direction and would be of great benefit, especially in case of ocular surface diseases. Drug delivery to the posterior segment of the eye can also be accomplished via SLNs and NLCs by proper adjustment of the formulation-related parameters (lipid constituents/surfactant(s) selection; tuning particles’ size into the desired nano range), which would be of great significance in the therapy of vision-threatening diseases. However, despite all the promising outcomes from conducted studies, the research progress has not been implemented into clinical application yet. Some of the challenges related to this matter include the possibility of developing reproducible batches of lipid nanoparticles, which exhibit sufficient colloidal stability during storage. In this regard, the implementation of quality-by-design (QbD) approach during the (pre)formulation stage is a feasible strategy, as it provides the possibility to obtain a final product with predictable quality attributes, which would benefit and facilitate nanocarriers’ subsequent commercialization [214]. Ocular toxicity is another critical issue to be considered during the development of ophthalmic formulations. According to the findings from the reviewed articles, SLNs and NLCs showed no level of toxicity (based on in vitro or in vivo studies), however, further studies are needed to evaluate their long-term toxicity, as well as their fate after application in vivo [215]. Regarding their clinical application approval, it is crucial to establish unified protocols evaluating their safety and effectiveness [107]. Based on the promising results from the conducted studies, it can be concluded that the potential of SLNs and NLCs should be fully deployed in the near future.

## Figures and Tables

**Table 1:** Alternative routes of ocular drug delivery.

Alternative Route	Specifics	Benefits	Limitations	References
Sub- conjunctival (SC)	SC route includes SC injections, administered in the lower or upper fornix, as well as instillation of SC implants; Clinical indications include corneal/scleral lesions, glaucoma, cytomegalovirus rhinitis.	Possibility to ensure high local drug concentration; Improved penetration of water-soluble drugs due to the bypassing of the corneal epithelium.	Conjunctival and choroidal blood/lymphatic flow; Temporary pain at the injection site; Local irritations.	[98,99]
Intracameral (IC)	Injections applied in the anterior chamber, often as a prevention of postoperative endophthalmitis after cataract surgery; Delivery of antibiotics, steroids, anesthetics.	Lower drug concentration needed; Decreased side effects vs. topical steroid application; Increased anesthesia during surgery when co-administered with topical anesthetics.	Potential complications, such as toxic anterior segment syndrome, corneal endothelial toxicity.	[100,101,102]
Transscleral	Drug delivery to the posterior segment of the eye; The sclera is thinnest around the equator, therefore, it is the preferred area for injection.	Obviates the corneal and conjunctival barrier; Less-invasive procedure compared to intravitreal injections.	Static barriers (sclera, choroid, retina) and dynamic barriers (choroidal blood flow) reduce drug bioavailability; Necessity of high doses.	[84,99,103]
Supra-choroidal (SC)	Drug injection under the choroid, targeting the following areas: choroid and retina; Microneedles have also been used for drug deposition into the SC space; Clinical indications include: posterior uveitis, macular edema.	Obviates the sclera and improves drug bioavailability within the choroid and retina; Effective for the delivery of small molecules; Lower risk of intraocular pressure spikes.	Choroidal circulation; Risk of occurrence of choroidal hemorrhage or detachment.	[99,104,105]
Intravitreal (IV)	Direct injection to the vitreous body targeting posterior eye segment; Drug delivery of vascular endothelial growth factor (VEGF) inhibitors, antibiotics, corticosteroids; IV injections are applied in the therapy of age-related macular degeneration, cytomegalovirus retinitis, diabetic macular edema, retinal vein occlusions.	Bypasses the BRB; Provides high local therapeutic concentration and prolonged drug levels; Reduced systemic side effects.	Repetitive instillations lead to serious ocular complications and patient non-compliance. Eye discomfort and pain were reported following IV injections.	[53,106]
Systemic/Oral	Drugs are administered orally or intravenously; Therapeutic applications include: scleritis, cytomegalovirus retinitis.	Acceptance by the patients.	Low bioavailability (<2%)— barrier role of BAB, BRB; Necessity of high doses, corresponding to increased risk of side effects.	[107]

**Table 2:** Recent progress of SLNs for ophthalmic application (5 years’ overview).

Composition	Drug/Disease	Method of Preparation	Physicochemical Characteristics	Results	References
Tripalmitin Tween 80 Glycerol	Econazole/ *Fungal keratitis*	Microemulsion method	Size 19.05 ± 0.28 nm PDI 0.21 ± 0.01 ζ potential −2.20 ± 0.10 mV EE = 94.18 ± 1.86%	Slow and controlled drug release (within 96 h); Improved antifungal activity; Enhanced bioavailability—drug concentration was above MIC within 3 h after application.	[153]
Precirol ATO 5 Pluronic F68 Stearyl amine	Natamycin/ *Fungal keratitis*	Hot emulsification-ultrasonication technique	Size 42 nm PDI 0.224 ζ potential 26 mV EE ≈ 85%	Prolonged drug release (within 8 h); Improved corneal penetration; Superior antifungal activity vs. free drug; Excellent ocular tolerability.	[154]
Compritol 888 ATO Stearic acid Tween 80 Soy lecithin	Isoniazid/ *Ocular tuberculosis*	Microemulsion method	Size 149.2 ± 4.9 nm PDI 0.15 ± 0.02 ζ potential −0.35 ± 0.28 mV EE = 65.2 ± 2.2%	Prolonged drug release (48 h); Enhanced corneal permeability (1.6 fold); Improved ocular bioavailability (4.2 fold) vs. drug solution.	[155]
Stearic acid Tween 80 Transcutol P	Clarithromycin/ *Bacterial endophthalmitis*	High-speed mixing and the ultrasonication method	Size 157 ± 42.4 nm PDI 0.13 ± 0.02 ζ potential −17.2 ± 3.1 mV EE = 81.3 ± 4.6	Sustained drug release (~80% in 8 h); Improved transcorneal permeation and bioavailability compared to drug solution.	[156]
Softisan 100 (Hydrogenated Coco-Glycerides) Suppocire NB (C10–C18 Triglycerides) Tween 80 Tegin O DOTAP DDAB	Sorafenib/ *Uveal melanoma*	Phase inversion temperature method	Size 127.85 ± 1.50 nm PDI 0.215 ± 0.014 ζ potential 20 mV EE= 75.0 ± 2.1%	Sustained drug release (less than 25% of encapsulated drug released after 72 h); Good physical stability, cytocompatibility and mucoadhesive properties of elaborated SLNs.	[157]
Compritol 888ATO PEG 400 Poloxamer 188 Phospholipon 90H	Atorvastatin/ *Age-related macular degeneration*	Hot high-pressure homogenization	Size 256.3 ± 10.5 nm PDI 0.26 ± 0.02 ζ potential −2.65 mV EE= 73.1 ± 1.52%	Improved bioavailability (8-fold in aqueous humor and 12-fold in vitreous humor) vs. free drug; Proven safety in corneal/retinal cell lines; Successful delivery to the retina, confirmed by intact fluorescein-labeled SLNs.	[158]
Com- pritol 888 ATO/Compritol HD5 ATO Pluronic F127	Betulinic acid (BA) derivatives H3, H5 and H7/ *Retinal diseases (diabetic retinopathy, age-related macular degeneration, choroidal neovascularization)*	Microemulsion method	Size 58.5± 9.8 nm PDI 0.246 ζ potential 6.45 ± 5.58 mV EE = 75.10%	Improved drug delivery and enhanced anti-oxidative efficacy of BA derivatives; Suppressed glutamate-induced ROS production/necrosis in human Müller cells.	[159]
Gelucire 44/14 Compritol ATO 888 Tween 80	Etoposide/ *Posterior segment-related diseases (e.g., age-related macular degeneration, diabetic retinopathy)*	Melt- emulsification and ultrasonication technique	Size 239.43 ± 2.35 nm PDI 0.261 ± 0.001 EE 80.96 ± 2.21%	Sustained etoposide concentration of etoposide in vitreous body for 7 days after IV injection Better toxicological profile vs. etoposide solution.	[160]
Stearic acid Sodium taurodeoxycholate Phosphati- dylcholine	Sutinib (Sb)/ *Retinal diseases* (*age-related macular degeneration, diabetic retinopathy, retinal vein occlusions)*	Microemulsion method	Size 140 nm PDI 0.20	Excellent tolerability profile based on in vivo study on 20 albino rabbits; After IV injections, Sb SLNs didn’t cause any abnormalities in ocular morphology in contrast to polymeric nanocapsules.	[161]
Chitosan Phospholipids (Lipoid S100) Glyceryl mono- stearate Tween 80 PEG 400	Methazolamide/ *Glaucoma*	Emulsion-solvent evaporation method	Size 247.7 ± 17.3 nm PDI ζ potential 33.5 ± 3.9 mV EE = 58.5 ± 4.5%	Prolonged drug release compared to drug solution; Excellent tolerability and marked reduction in IOP vs. uncoated methazolamide SLNs.	[162]
Compritol 888 ATO Pluronic F68 Tween 80 Glycerol	Δ^9^ -Tetrahydrocannabinol-valine-hemisuccinate/ *Glaucoma*	Ultrasonication	Size 287.80 ± 7.35 nm PDI 0.29 ± 0.01 EE = 93.57 ± 4.68%	Greater reduction in the IOP with respect to intensity and duration compared to pilocarpine/timolol maleate eye drops; High drug concentration in the iris/ciliary body and choroid/ retina.	[163]

Legend: DDAB—Didodecyldimethylammonium bromide; DOTAP—Dioleoyl-trimethylammonium–propane chloride; EE—Entrapment efficiency; IOP—Intraocular pressure; MIC—Minimum inhibitory concentration; PDI—Polydispersity index; ROS—Reactive oxygen species.

**Table 3:** Recent progress of NLCs for ophthalmic application (5 years’ overview).

Composition	Drug/*Disease*	Method of Preparation	Physicochemical Characteristics	Results	References
Glycerol monostearate 40–55 Soy lecithin Compritol 888 ATO Cholesterol Capryol 90 Miglyol 812 N Kolliphor P 407 Kolliphor P 188 α-Tocopherol-PEG	Lactoferrin/ *Keratoconus*	Double emulsion/ solvent evaporation method.	Size 119.45 ± 11.44 nm PDI 0.151 ± 0.045 ζ potential 17.50 ± 2.53 mV EE ≈ 75%	Controlled release profile; Good physical stability (up to 3 months); Muco-adhesive properties (for at least 240 min); Ocular tolerability.	[193]
Labrafac lipophile WL1349 Cholesterol Tween 80	Dexamethasone (DXM)/ *Dry Eye Disease*	Solvent diffusion method	Size 19.51 ± 0.5 nm PDI 0.08 ζ potential 9.8 mV EE = 99.6 ± 0.5%	Cellular internalization in HCECs and corneal distribution in ex vivo porcine cornea; Significant reduction in inflammatory cytokines (MMP-9, IL-6 and TNF-α) related to DED pathogenesis vs. free DXM.	[194]
Precirol ATO5 Capryol PGMC Stearylamine Tween 80 Poloxamer 188	Rapamycin/ *Corneal alkaline burn injury*	Emulsification solvent diffusion and evaporation method	Size 216 ± 40 nm ζ potential 14 ± 2.6 mV EE = 97.66 ± 0.57%	Improved fibroblast uptake of encapsulated cargo via NLCs (1.5 times); Superior in vivo corneal healing properties of NLCs vs. control groups.	[195]
Stearic acid, oleic acid Poloxamer 407	Itraconazole/ *Fungal keratitis*	High-speed homogenization technique	Size 150.67 nm ζ potential −28 mV EE = 94.65%	Ocular safe formulation according to HET−CAM test; Enhanced antifungal activity of the NLCs compared to commercial eye drops.	[196]
PrecirolATO 5,Castor oil, Span 80, mPEG-2K-DSPE sodium salt Poloxamer 188, Tween 80, glycerin	Natamycin/ *Fungal keratitis*	High-pressure homogenization	Size 241.96 nm, PDI 0.406 EE = 95.35%	Improved in vitro transcorneal permeation and flux of formulated NT compared to drug suspension.	[197]
Glycerin monostearate Miglyol 812 N Solutol HS 15 Gelucire 44/14 Soy lecithin	Dasatinib (DAS)/ *Corneal neovascularization*	Melt-emulsification method	Size 78.53 ± 0.36 nm PDI 0.21 ± 0.01 ζ potential −29.6 ± 1.0 mV EE = 97.71% ± 0.89%	Enhanced solubility of DAS (1200-fold) after inclusion in NLCs; Inhibition of the development of CNV and associated corneal pathological alterations in a mouse model of CNV.	[198]
Monolaurin Capryol-90 Cremophor RH40 Transcutol P Glycerin	Sorafenib/ *Corneal neovascularization*	Microemulsion method	Size 111.87 ± 0.93 nm PDI 0.15 ± 0.01 ζ potential−0.35 ± 0.08 mV EE = 99.20 ± 0.86%	Excellent ocular tolerability (in vivo test on rabbits), non-toxic in HCEC; Approximately 6.7- and 1.3-fold higher drug concentrations in rabbit cornea and conjunctiva vs. free drug.	[199]
Compritol 888 ATO Apifil (PEG-8 beeswax) Miglyol 812N Labrasol, Kolliphor EL Cremophor RH60	Dexamethasone/ *Ophthalmic inflammatory diseases, severe uveitis*	Ultrasonication method	Size 92.18 ± 0.49 nm PDI 0.12 ± 0.02 ζ potential −7.62 ± 0.26, EE = 88.31%	Good ocular tolerability; Ability to penetrate across the cornea; High concentration of NLCs in the stroma, according to porcine corneal penetration study.	[171]
Capmul MCM C10 Soya lecithin Captex 200 P Transcutol P Polysorbate 80 Stearylamine	Triamcinolone acetonide/ *Uveitis*	Hot microemulsion method	Size 198.95 ± 12.82 nm PDI 0.326 ± 0.04 ζ potential 35.8 ± 1.94 mV EE = 88.14 ± 3.03 %	Sustained drug release (84% within 24 h); Ex vivo corneal permeation of 51%; Biocompatible and ocular tolerable formulation (HET-CAM test).	[200]
Cholesterol Stearic acid Stearylamine Oleic acid Labrafil M 1944 Tween 80	Vancomycin (VMC)/ *Bacterial endophthalmitis*	Cold homogenization technique	Size 96.40 ± 0.71 nm PDI 0.352 ± 0.011 ζ potential 29.7 ± 0.47 mV, EE = 74.80 ± 4.30%	Improved transcorneal penetration; Biocompatible, non-irritant formulation (in vitro RBC hemolytic assay); Enhanced (3-fold) intravitreal VMC concentration after topical application compared to drug solution.	[201]
Miglyol 812 Compritol 888 ATO Lutrol F68	Palmitoylethanolamide (PEA)/ *Retinal diseases* *(diabetic retinopathy, glaucoma)*	High shear homogenization	Size 208.6 ± 10.2 nm PDI 0.18 ζ potential > 20 mV	Improved ocular bioavailability: 40% and 100% higher PEA levels in vitreous body and retina compared to free drug.	[202]
Glyceryl monostearate Labrafil M 2125 CS Tween 80 Transcutol HP Chitosan	5-Fluorouracil (5-FU)/ *Diabetic* *retinopathy*	Melt emulsification-ultrasonication method	Size 163.2 ± 2.3 nm PDI 0.28 ± 1.52 ζ potential 21.4 ± 0.5 mV EE = 85.0 ± 0.2 %	Higher and sustained 5-FU release vs. free drug; Non-irritant formulations; Antiangiogenic effect confirmed by in vivo study in a diabetic retinopathy rat model.	[181]
Capryol 90 Softisan 100 Tween 80	Diosmin/ *Diabetic* *retinopathy*	Melt emulsification method and ultrasonication	Size 83.58 ± 0.77 nm PDI 0.263 ± 0.067 ζ potential −18.5 ± 0.60 mV EE = 99.53± 2.50	Very good physical stability of NLCs up to 60 days; Cytocompatibility assessed on ARPE-19 cells, Cytoprotective effects.	[203]
Compritol 888 ATO Miglyol 812 Lutrol F68	Mangiferin (MNG)/ *Oxidative stress related diseases, macular degeneration, diabetic retinopathy*	High shear homogenization and ultrasound	Size 148.9 ± 0.1 nm PDI 0.21 ± 0.02 ζ potential −23.5 ± 0.2 mV, EE ≈ 92%	Higher antioxidant activity of MNG NLCs vs. free compound according to ORAC assay; Non-irritant formulations according to HET−CAM Assay.	[204]
Glyceryl monostearate Castor oil Poloxamer 188	Brimonidine/ *Glaucoma, ocular hypertension*	High shear homogenization	Size 151.97 ±1.98 nm PDI 0.230 ± 0.01 ζ potential −44.2 ± 7.81 mV EE = 83.631 ± 0.495%	Improved permeability compared to analogous model SLNs; Highest reduction in the IOP in rabbits (vs. SLNs and free drug).	[172]
Captex 200P (propylene glycol dicaprate) Soya lecithin Capmul^®^ MCM C10 (glyceryl monocaprate) Tween 80 Transcutol P Stearylamine Captex 200P	Brinzolamide (Brla) Latanoprost (Ltp)/ *Glaucoma*	Hot microemulsion method	Size165.28 ± 2.36 nm PDI 0.31 ± 0.015 ζ potential 35.33 ± 0.37 mV EE = 97.5 ± 2.16%	Adequate transcorneal permeation (Brla and Ltp levels after 24 h were ≈82% and ≈84%, respectively); Effective reduction of IOP in rats’ eyes with laser-induced glaucoma.	[192]

Legend: ARPE—Human retinal pigment epithelial cell line, CNV—Corneal neovascularization, DED—Dry eye disease, HCEC—Human corneal epithelial cell lines, HET−CAM—Hen’s egg test on chorioallantoic membrane, IL-6—Interleukin-6, MMP—Matrix metalloproteinases, mPEG-2K-DSPE sodium salt—N-(Carbonyl-methoxypolyethylenglycol-2000)-1,2-distearoyl-sn-glycero-3-phosphoethanolamine sodium salt, ORAC—Oxygen radical absorbance capacity, TNF-α—Tumor necrosis factor α.

## Data Availability

Data sharing not applicable.

## Supplementary Materials

The following supporting information can be downloaded at: https://www.mdpi.com/article/10.3390/ph16030474/s1, Table S1. Solid lipid nanoparticles and nanostructured lipid carriers in clinical trials (terminated studies and studies with unknown status are excluded).
