# Supplementary material for: Recent Progress of Solid Lipid Nanoparticles and Nanostructured Lipid Carriers as Ocular Drug Delivery Platforms
Source: Pharmaceuticals (Basel). 2023 Mar 22;16(3):474. doi: 10.3390/ph16030474 (PMC10058782; doi:10.3390/ph16030474)
Supplement: Supplementary file 1 [file pharmaceuticals-16-00474-s001.zip › pharmaceuticals-2251665-supplementary.docx]

***Supplementary file***

***for***

Recent progress of solid lipid nanoparticles and nanostructured lipid carriers as ocular drug delivery platform

Table S1. Solid lipid nanoparticles and nanostructured lipid carriers in clinical trials (terminated studies and studies with unknown status are excluded)

| **Clinical trial Identifier** | **Study Title** | **Nanocarrier** | **Drug** | **Indication** | **Status** |
| --- | --- | --- | --- | --- | --- |
| NCT03823040 | Clinical assessment of oxiconazole nitrate solid lipid nanoparticles loaded gel | SLNs | Oxiconazole  nitrate | Tinea | Phase 1 completed |
| NCT04803500 | Simvastatin around immediate implant | SLNs/gel | Simvastatin | Immediate implant placement  Bone regeneration  Alveolar bone resorption | Phase 2 completed |
| NCT05267899 | A phase I first in human study to evaluate the safety, tolerability, and pharmacokinetics of wgi-0301 in patients with advanced solid tumors | Lipid nanoparticle suspension | WGI-0301 | Advanced solid tumors | Recruiting |
| NCT03739931 | Dose escalation study of mrna-2752 for intratumoral injection to participants in advanced malignancies | Lipid nanoparticle | mRNA-2752/ mRNA-2752+  Durvalumab | Dose escalation: relapsed/refractory solid tumor malignancies or lymphoma  Dose expansion: triple negative breast cancer, hnscc, non-hodgkins, urothelial cancer, immune checkpoint refractory melanoma, and nsclc lymphoma | Recruiting |
| NCT04675996 | First-in-human study of int-1b3 in patients with advanced solid tumors | Lipid nanoparticle | INT-1B3 | Solid tumor | Recruiting |
| NCT05497453 | A phase 1/2 study to evaluate otx-2002 in patients with hepatocellular carcinoma and other solid tumor types known for association with the  MYC oncogene | Lipid nanoparticle | OTX-2002/  OTX-2002 +Tyrosine kinase inhibitor One/  OTX-2002 +Tyrosine kinase inhibitor Two/ OTX-2002 +  Checkpoint Inhibitor, Immune | Hepatocellular carcinoma  Solid tumor Hepatocellular carcinoma non-resectable Hepatocellular carcinoma recurrent Hepatocellular cancer  Liver cancer  Liver, cancer of, non-resectable | Recruiting |
| NCT05370040 | THEMBA II T-Cell Vaccine: Vaccination with saRNA COVID-19 vaccines | NLCs | AAHI-SC2 Vaccine/  AAHI-SC3 Vaccine/  EUA or approved vaccine | COVID-19 | Recruiting |
