# Supplementary material for: Recent Progress of Solid Lipid Nanoparticles and Nanostructured Lipid Carriers as Ocular Drug Delivery Platforms
Source: Pharmaceuticals (Basel). 2023 Mar 22;16(3):474. doi: 10.3390/ph16030474 (PMC10058782; doi:10.3390/ph16030474)
Supplement: Supplementary file 1 [file pharmaceuticals-16-00474-s001.zip › pharmaceuticals-2251665-supplementary.pdf]

Supplementary file

for

Recent progress of solid lipid nanoparticles and nanostructured lipid carriers as ocular drug delivery platform

Table S1. **Solid lipid nanoparticles and nanostructured lipid carriers in clinical trials (terminated studies and studies with unknown status are excluded)**

Clinical trial Identifier	Study Title	Nanocarrier	Drug	Indication	Status
NCT03823040	Clinical assessment of oxiconazole nitrate solid lipid nanoparticles loaded gel	SLNs	Oxiconazole nitrate	Tinea	Phase 1 completed
NCT04803500	Simvastatin around immediate implant	SLNs/gel	Simvastatin	Immediate implant placement<br>Bone regeneration<br>Alveolar bone resorption	Phase 2 completed
NCT05267899	A phase I first in human study to evaluate the safety, tolerability, and pharmacokinetics of wgi-0301 in patients with advanced solid tumors	Lipid nanoparticle suspension	WGI-0301	Advanced solid tumors	Recruiting
NCT03739931	Dose escalation study of mrna-2752 for intratumoral injection to participants in advanced malignancies	Lipid nanoparticle	mRNA-2752/ mRNA-2752+ Durvalumab	Dose escalation: relapsed/refractory solid tumor malignancies or lymphoma<br>Dose expansion: triple negative breast cancer, hnscc, non-hodgkins, urothelial cancer, immune checkpoint refractory melanoma, and nsclc lymphoma	Recruiting
NCT04675996	First-in-human study of int-1b3 in patients with advanced solid tumors	Lipid nanoparticle	INT-1B3	Solid tumor	Recruiting
NCT05497453	A phase 1/2 study to evaluate otx-2002 in patients with hepatocellular carcinoma and other solid tumor	Lipid nanoparticle	OTX-2002/ OTX-2002 +Tyrosine kinase inhibitor One/ OTX-2002 +Tyrosine kinase	Hepatocellular carcinoma Solid tumor Hepatocellular carcinoma non-resectable Hepatocellular carcinoma recurrent Hepatocellular cancer	Recruiting

	types known for association with the MYC oncogene		inhibitor Two/ OTX-2002 + Checkpoint Inhibitor, Immune	Liver cancer<br>Liver, cancer of, non-resectable	
NCT05370040	THEMBA II T-Cell Vaccine: Vaccination with saRNA COVID-19 vaccines	NLCs	AAHI-SC2 Vaccine/ AAHI-SC3 Vaccine/ EUA or approved vaccine	COVID-19	Recruiting
